# Association of Steroids Use with Survival in Patients Treated with Immune Checkpoint Inhibitors: A Systematic Review and Meta-Analysis

**DOI:** 10.3390/cancers12030546

**Published:** 2020-02-27

**Authors:** Fausto Petrelli, Diego Signorelli, Michele Ghidini, Antonio Ghidini, Elio Gregory Pizzutilo, Lorenzo Ruggieri, Mary Cabiddu, Karen Borgonovo, Giuseppina Dognini, Matteo Brighenti, Alessandro De Toma, Erika Rijavec, Marina Chiara Garassino, Francesco Grossi, Gianluca Tomasello

**Affiliations:** 1 Medical Oncology Unit, ASST Bergamo Ovest, 24047 Treviglio (BG), Italy; mary_cabiddu@asst-bgovest.it (M.C.); karen_borgonovo@asst-bgovest.it (K.B.); 2 Thoracic Oncology, Medical Oncology Department, Fondazione IRCCS Istituto Nazionale dei Tumori, 20133 Milan, Italy; diegosignorelli@yahoo.it (D.S.); alessandro.detoma@istitutotumori.mi.it (A.D.T.); marina.garassino@istitutotumori.mi.it (M.C.G.); 3 Medical Oncology Unit, Fondazione IRCCS Ca’ Granda, Ospedale Maggiore Policlinico, 20122 Milano, Italy; mghido@hotmail.it (M.G.); erika.rijavec@policlinico.mi.it (E.R.); francesco.grossi@policlinico.mi.it (F.G.); 4 Medical Oncology Unit, Casa di Cura Igea, 20126 Milano, Italy; antonioghidini@hotmail.com; 5 Medical Oncology Unit, Niguarda Cancer Center, Grande Ospedale Metropolitano Niguarda, 20162 Milano, Italy; eliogregory.pizzutilo@ospedaleniguarda.it (E.G.P.); lorenzo.ruggieri@ospedaleniguarda.it (L.R.); gianluca.tomasello@gmail.com (G.T.); 6 Medicine Unit, ASST Bergamo Ovest, 24047 Treviglio (BG), Italy; g.dognini@gmail.com; 7 Oncology Unit, ASST Cremona, 26100 Cremona, Italy; mbrighenti20@gmail.com

**Keywords:** immunotherapy, immune-related adverse events, prognosis, steroids, meta-analysis

## Abstract

Immune checkpoint inhibitors (ICIs) can elicit toxicities by inhibiting negative regulators of adaptive immunity. Sometimes, management of toxicities may require systemic glucocorticoids. We performed a systematic review and meta-analysis of published studies to evaluate the correlation between steroids use, overall survival (OS), and progression-free survival (PFS) in cancer patients treated with ICIs. Publications that compared steroids with non-steroid users in cancer patients treated with ICIs from inception to June 2019 were identified by searching the EMBASE, PubMed, SCOPUS, Web of Science, and Cochrane Library databases. The pooled hazard ratios (HRs) and 95% confidence intervals (CIs) were calculated using a random-effects model. Patients (studies, *n* = 16; patients, *n* = 4045) taking steroids were at increased risk of death and progression compared to those not taking steroids (HR = 1.54, 95% CI: 1.24–1.91; *p* = 0.01 and HR = 1.34, 95% CI: 1.02–1.76; *p* = 0.03, respectively). The main negative effect on OS was associated with patients taking steroids for supportive care (HR = 2.5, 95% CI 1.41–4.43; *p* < 0.01) or brain metastases (HR = 1.51, 95% CI 1.22–1.87; *p* < 0.01). In contrast, steroids used to mitigate adverse events did not negatively affect OS. In conclusion, caution is needed when steroids are used for symptom control. In these patients, a negative impact of steroid use was observed for both OS and PFS.

## 1. Introduction

Immune checkpoint inhibitors (ICIs) have improved patient outcomes in different tumors. The anti-Cytotoxic T-Lymphocyte Antigen 4 (CTLA-4) antibody ipilimumab and the anti-Programmed Death 1 (PD-1) drugs nivolumab and pembrolizumab have radically changed the therapeutic scenario in melanoma. The anti-Programmed Death Ligand 1 (PDL-1) durvalumab is the gold standard in unresectable locally advanced PDL-1 positive (tumor proportion score > 1%) Non-Small Cell Lung Cancer (NSCLC) as maintenance treatment after definitive chemoradiotherapy. In advanced NSCLC without EGFR or ALK aberrations, immunotherapy alone is the standard treatment in second line and, in PDL-1 strong positive (tumor proportion score > 50%) tumors, in first line [1]. The combination of chemotherapy plus immunotherapy is a new option in first line in advanced NSCLC, regardless of PDL-1 expression [2,3,4].

Corticosteroids have immunosuppressive properties through pleiotropic activities on T cell activation, differentiation, and migration [5], suppressing IL-2 mediated activation of effector T cells [6] and increasing regulatory T-cells [7]. Steroids can modify microbiome [8] and stimulate M2 macrophage polarization [9]. Because of their immunosuppressive properties, corticosteroids are both the principal treatment of immune-related adverse events due to ICIs [10] and an exclusion criterion for ICIs clinical trials; a threshold of ≥10 mg of prednisone equivalent daily is the usual cutoff [11,12]. Doses ≥ 10 mg of prednisone daily are related to increasing infection rates in patients chronically treated with steroids [13], and are therefore considered immunosuppressive. However, corticosteroids are often used at higher doses as palliative treatment for cancer-related symptoms such as dyspnea, fatigue, and symptomatic brain metastases [14,15,16].

The role of steroids administration during treatment with ICIs is still debatable. Their use, also at high doses, to manage immune-related adverse events did not affect ICIs efficacy in patients with melanoma [17] and NSCLC [18]. However, the early daily administration of ≥10 mg of prednisone equivalent at the time of ICIs initiation was related to poor outcomes in patients with NSCLC in some retrospective analysis [19,20,21]. Furthermore, a recent paper confirmed the worse outcomes in NSCLC patients treated with ICIs if doses ≥ 10 mg of prednisone equivalent were administered within 24 h of ICIs initiation. However, the detrimental corticosteroids effect was evident only in patients who were on steroids therapy because of cancer-related palliative indications; doses ≥ 10 mg of prednisone for cancer-unrelated indications, such as treatment of autoimmune disease, chronic obstructive pulmonary disease flare, and prophylaxis for hypersensitivity reactions, were not associated with worse outcomes in comparison with less than 10 mg of prednisone or no steroids administration [22].

A prospective randomized controlled clinical trial of steroids dose reduction or interruption is difficult to conduct. To better define their role during treatments with ICIs alone, we performed a systematic review and meta-analysis.

## 2. Materials and Methods

The search process followed the Preferred Reporting Items for Systematic Reviews and Meta-analyses guidelines. The outcomes of the present meta-analysis were reported according to the Meta-analysis of Observational Studies in Epidemiology criteria [23].

### 2.1. Search Strategy and Inclusion Criteria

A systematic search of electronic databases was conducted to identify studies that analyzed outcome in advanced cancer patients treated with ICIs and steroids. Published articles that compared steroids with non-steroid users in cancer patients treated with ICIs from inception to June 2019 were identified by searching the EMBASE, PubMed, SCOPUS, Web of Science, and Cochrane Library databases. Hand searches were also performed to identify other potentially eligible studies. The following keywords were used as search terms: (steroid or glucocorticoid or corticosteroid) and survival and (pd-1 or pd-l1 or ctla-4 or “immune checkpoint inhibitors”).

Three independent authors (F.P., G.T., and D.S.) performed the searches and assessed study eligibility. Prospective or retrospective studies, published in English language, comparing overall survival (OS) and progression-free survival (PFS) between ICIs + steroids use (intervention group) and ICIs use alone (comparator group) in cancer patients were selected. Exclusion criteria applied during the selection process were as follows: (1) conference abstracts; (2) reviews, editorials, comments, and letters; (3) case reports; (4) studies not reporting the survival outcome of steroid user patients; (5) lack of information regarding a comparator group; and (6) insufficient data to extract hazard ratios (HRs) and 95% confidence intervals (CIs). The name of the institution or database included in the final set of eligible studies was reviewed. When multiple studies were based on the same dataset, the one with the longest-duration study period and the largest number of patients was selected. The study selection process was assessed independently by a third investigator (FG).

### 2.2. Data Extraction

The data were extracted independently by three authors (FP, GT, and AG). When discrepancies occurred, the authors discussed to reach a consensus. Author, year of publication, type of studies, diseases included, median follow up, dose and steroid regimens, number of steroid users, and reason for steroids intake were extracted from publications. Risk of bias assessment was conducted independently by three authors (FP, AG, and MG). For randomized studies, the Cochrane Collaboration’s tool for assessing the risk of bias in randomized trials was used. Since almost all of the included studies were non-randomized observational studies, the Risk of Bias Assessment tool for Nonrandomized Studies was used to assess the following six domains: the selection of participants; confounding variables; intervention measurement; blinding of the outcome assessment; incomplete outcome data; and selective outcome reporting [24,25]. Regarding potential discrepancies among the three authors, a consensus was obtained after further review and discussion with a senior author (FG). Quality of paper was evaluated through the Nottingham–Ottawa-Scale (NOS) for observational studies [26]. The total scores ranged from 0 (worst) to 9 (best) for cohort studies, with a score of at least seven indicative of high quality.

### 2.3. Statistical Analysis

The primary outcome of interest was OS and the secondary endpoint was PFS. The HRs and 95% CIs from each study were either extracted directly from original papers or calculated using Kaplan–Meier curves based on the method of Tierney et al. HRs were calculated using a random-effects model with the inverse variance method. Cochrane Q tests and the I^2^ index were used to evaluate heterogeneity. Funnel plots with Egger’s regression tests were used to examine publication bias. Additional stratified OS analyses were performed to compare results from mono- and multi-center studies, retrospective and prospective studies, reason for steroid use (e.g., supportive care vs. brain metastases vs. adverse events [AEs]), number of patients [>100 vs. <100]), type of disease (NSCLC vs. melanoma vs. others), type of agent (anti-PD-(L)1 vs. anti-CTLA-4 vs. combinations, if data were available), type of analysis (uni- vs. multi-variate), and quality of paper (NOS ≥ 7 vs. < 7) were performed. RevMan software (ver. 5.3; Cochrane Collaboration, Copenhagen, Denmark) was used for all pooled analyses.

## 3. Results

**Figure 1:**
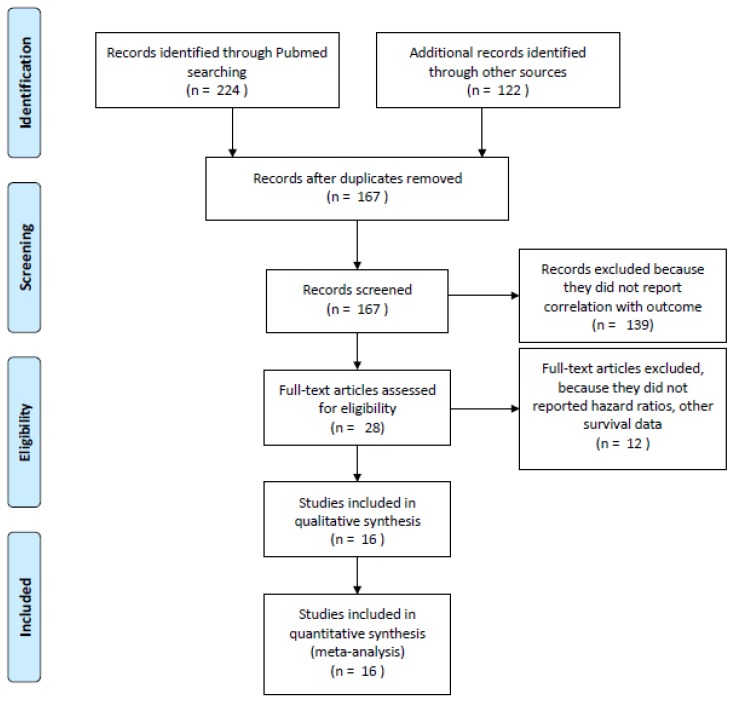
Flow diagram of included studies.

In total, 346 potentially relevant citations were reviewed (Figure 1). Ultimately, 16 studies published from 2009 to 2019 that reported OS and/or PFS data were included in the final analysis [17,19,20,21,22,27,28,29,30,31,32,33,34,35,36,37]. The total number of patients included was 4045 ranging from 45 to 1025 patients per study (median, 151). The major characteristics are shown in Table 1. All but one (Weber 2009) were retrospective studies. Seven studies included patients with melanoma; the remaining *n* = 9 studies included NSCLCs (*n* = 7) or various histotypes (*n* = 2). Stages were mixed (III–IV) with *n* = 11 studies including only metastatic disease. According to the different study, patients received ICIs (nivolumab, pembrolizumab, atezolizumab, durvalumab, and ipilimumab) alone or in combination. In most studies (*n* = 9), steroids were administered for supportive care reasons; in six studies, steroids were used following immune-related adverse events (IrAEs). The quality of paper expressed by the NOS scale ranged from 4 to 8, with almost all studies (94%) of sufficient to high quality (mean NOS scale scores: 6.69).

### 3.1. Meta-Analysis of OS 

**Figure 2:**
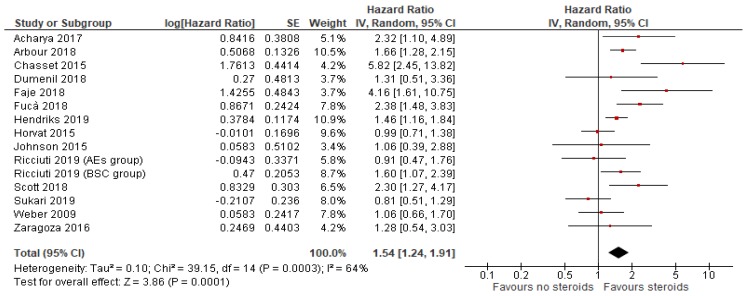
Overall survival comparing use or not of steroids concomitant to immune checkpoint in patients with cancer.

OS data were available in *n* = 14 studies. Because the heterogeneity test showed a high level of heterogeneity (I^2^ = 64%, *p* < 0.001) between the studies, a random-effects model was used for the analysis. Overall prognosis of patients receiving steroids for any reason during treatment with ICIs was significantly worse (HR = 1.54, 95% CI: 1.24–1.91; *p* = 0.0001; Figure 2). 

### 3.2. Meta-Analysis of PFS

**Figure 3:**
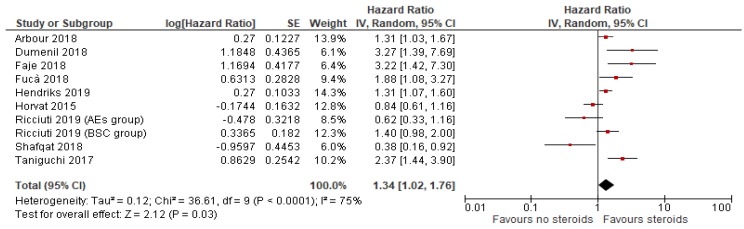
Progression-free survival comparing use or not of steroids concomitant to immune checkpoint in patients with cancer.

PFS data were available in *n* = 9 studies with high heterogeneity (I^2^ = 75%, *p* < 0.001), thus a random-effects model was used for the analysis. Concomitant use of steroids in patients treated with ICIs was associated with a 34% higher risk of progression or death (HR = 1.34; 95% CI: 1.02–1.76; *p* = 0.03) (Figure 3).

### 3.3. Subgroup Analysis 

A further subgroup analysis was performed according to the following variables: number of patients (≥100 or <100), type of study (multi- vs. mono-centric), study quality (NOS score ≥7 vs. NOS score < 7), type of agent, and type of disease (NSCLC vs. melanoma) and found no significant differences that would confirm a worse prognosis associated with steroid use. However, when the reason for using steroids was split by supportive care vs. brain metastases, the supportive care subgroup was associated with a worse prognosis (HR = 2.51, 95% CI 1.41–4.43; *p* < 0.01). Conversely, in patients taking steroids for IrAEs, the outcome was not compromised (Table 2).

### 3.4. Publication Bias

**Figure 4:**
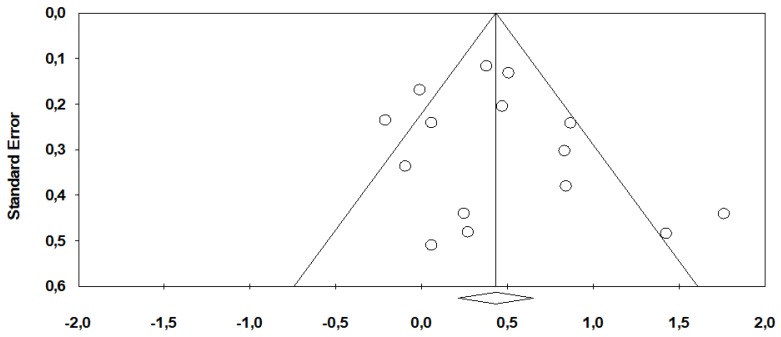
Funnel plots showing log hazard ratios and standard errors for overall survival.

There was no publication bias in the overall pooled results (*p* = 0.18 and *p* = 0.20 for OS pooled analysis through Begg’s and Egger’s test, respectively) (Figure 4).

## 4. Discussion

ICIs can elicit toxicities by inhibiting negative regulators of adaptive immunity. Usually, events of mild intensity do not require specific treatments but supportive care only. When more severe events develop, moderate to high-dose systemic glucocorticoids (generally prednisone 1 mg/kg or equivalent or intravenous formulations) are needed. Metastatic cancer patients may also need steroids for symptoms control such as dyspnea, pain, brain edema, and fatigue or for concomitant autoimmune diseases. Registered trials of ICIs used to exclude patients with pre-existing steroids use at equivalent doses greater than 10 mg of prednisone. Therefore, its potential detrimental effect on efficacy is currently unknown.

We performed a systematic review and meta-analysis of all published studies where outcome of corticosteroid user patients treated with immunotherapy was compared with those not assuming or using steroids at lower doses (inferior to 10 mg equivalent of prednisone). 

We found that patients taking steroids for any reason were at increased risk of death and progression compared to those not using steroids (HR = 1.54, *p* < 0.01 and HR = 1.34, *p* = 0.03, respectively). In subgroup analysis, the greatest negative effect on prognosis was evident in patients taking steroids for supportive care (e.g., disease-related symptoms), where the risk of death was more than doubled, and for brain metastases, where the risk of death was similar to the whole population and increased by 50%. Conversely, the effect of steroids administered to mitigate AEs did not seem to negatively affect OS; this finding was similar in NSCLC and melanoma. Similar results were presented by Ricciuti et al., where steroids’ detrimental effect appeared to be linked to the poor-prognosis subgroup of patients who received corticosteroids for palliative indications [22]. This may be associated with a larger number of patients with poor performance status or brain metastases where steroids are provided for cancer-related palliation. A potential association with better prognosis in patients reporting immune-related adverse events has been described [38,39], thus balancing the negative effect of steroid use. Specifically, in a pooled analysis of 28 studies where the OS of patients experiencing IrAEs was compared with that of patients without AEs, the risk of death was reduced by 50% in the IrAE group (Petrelli, personal communication).

The negative effect of steroids on survival in patients receiving ICIs appears intuitive. In preclinical models, dexamethasone whether given alone or in combination with anti-PD-1 therapy resulted in significant reductions of circulating CD4+ T cells. Absolute numbers of circulating CD8+ T cells also displayed similar significant trends with dexamethasone treatment. In the same experimental model, mice that received anti-PD-1 therapy alone experienced significantly longer tumor doubling times, thus delaying tumor growth compared to control group. Conversely, dexamethasone alone and anti-PD-1 + dexamethasone combination treatment group displayed a similar effect on tumor volume [40]. Another explanation of the way steroids impairs function of activated T lymphocytes is with the enhancing expression of PD-1 on T-cells [41]. More in general glucocorticoids induce apoptosis in hematological cells, thus supporting their use as therapeutic agents for leukemias, lymphomas, and myeloma [42]. Patients taking steroids at start of immunotherapy can so hamper the immune cascade, preventing the activation of an effective antitumor immune response.

Our paper has some intrinsic limitations but may provide an important clinical message to oncologists. First, this is a meta-analysis of mainly retrospective studies, where imbalance in prognostic factors may have led to negative association of steroids with outcome. Second, in many trials, type, dose, and duration of steroids used are unknown, and thus a correlation with timing and intensity of exposure was not possible. Third, papers include patients treated with anti-PD-(L)1 agents or ipilimumab for various cancers and a subgroup analysis was not possible. Fourth, the average effect was likely driven by negative prognostic factors and palliation indication for steroids in many cancer patients. Brain metastases or higher burden of thoracic or bone disease conditioning respiratory symptoms or pain seems to represent the primary indications for steroids in these studies. In addition, more advanced age, anorexia/weight loss, and poor performance status may have weighted as bad prognostic factors in steroid’s cohort and may have influenced the final analysis of OS. Finally, median follow up was relatively short or not reported in many publications, thus results could have been different if prolonged observation of events were performed by the authors. However, this meta-analysis is the first systematic collection and pooling of all data regarding the association of steroids use and prognosis during treatment with ICIs. Despite the overall results being derived from small single-center studies, it reassures the use of steroids during treatment with ICIs and highlights that low systemic dose of steroids used to manage AEs may not affect survival. On the contrary, symptomatic patients requiring steroids at the start of ICIs may require a different treatment approach (e.g., chemotherapy) or a rapid tapering of steroids before commencing immunotherapy.

## 5. Conclusions

In summary, even though high-grade immune-related toxicities necessitate corticosteroid therapy for improvement, use of steroids in these cases seems not to reduce OS in cancer patients treated with ICIs and may be safely administered without compromising outcome [10,43]. Conversely, more caution is needed for metastatic patients where steroids are used for reasons different from AEs (e.g., disease-related symptoms or brain metastases) and a detrimental effect on survival is likely. In these cases, discussing different treatment options (e.g., chemotherapy or radiotherapy) may avoid futile treatment while delaying treatment with ICIs until the symptom conditions are ameliorated and controlled without systemic steroids.

## Figures and Tables

**Table 1:** Characteristics of included studies.

Author/Year	N of Pts (Total for Study)	Type of Study/Median FU (Months)	Disease	Stage %	ICIs Used	Steroids Used/ N of Pts	Duration/Dose mg (*p* Equivalent)	Reason for Steroid Use	HR (95% CI) for OS	HR (95% CI) for PFS	Type of Analysis	Quality (NOS)/ Risk of Bias
**Acharya/2017 [33]**	72	Retrospective/8.9	Melanoma	IV (100)	NIVO, PEMBRO, IPI, anti-BRAF-MEK	DEX (90%)/21	NR/25–50 mg (DEX)	BMs (100%)	2.32 (1.1–4.80) *	-	MVA	6/low
**Arbour/2018 [19]**	640	Retrospective/NR	NSCLC	IV (100)	PEMBRO, NIVO, ATEZO or DURVA (100%)	NR/90	1–30 days before and at start of ICIs/ >10 mg vs. <10 mg	BMs (17%), BSC (83%)	1.66 (1.28–2.16) *	1.31 (1.03–1.67) *	MVA	7/low
**Chasset/2015 [32]**	45	Retrospective/21.9	Melanoma	III–IV	IPI	PDN, methyl-P/12	baseline/0.2 to 1.2 (mean 0.6) mg/kg	BMs (16%), BSC (84%)	5.82 (2.45–13.8) *	-	UVA	7/low
**Dumenil/2018 [34]**	67	Retrospective/NR	NSCLC	IIIB–IV	NIVO	NR/10	1st cycle of ICI/10–40 mg die in 5 patients NR in other 5 pts	BMs (100%)	1.31 (0.51–3.38)	3.27 (1.39–7.69) *	MVA	6/low
**Faje/2018 [35]**	98	Retrospective/NR	Melanoma	IV (100)	IPI	NR (high vs. low dose)/69	NR/22 mg vs. 5 in high vs. low dose	IrAEs (100%)	4.16 (1.61–14.28) *^	3.22 (1.42–8.33) * TTF ^	MVA	6/low
**Fuca’/2019 [21]**	151	Retrospective/28.6	NSCLC	IV (100)	anti-PD-1/PD-L1/anti-PD-L1 + anti-CTLA-4	NR/35	1–28 days after start of ICI/median 280 mg (range, 20–875 mg)	BSC (54%), NR (35%), IrAEs (11%)	2.38 (1.48–3.83) *	1.88 (1.08–3.28) *	MVA	8/low
**Hendriks/2019 [37]**	1025	Retrospective/15.8	NSCLC	Advanced	anti-PD-1/PD-L1	NR/141	start of ICI/NR	BMs (100%)	1.46 (1.16–1.84) *	1.31 (1.07–1.62) *	MVA	8/low
**Horvat/2015 [17]**	298	Retrospective/NR	Melanoma	III–IV	IPI	NR/103	NR/NR	IrAEs (100%)	0.99 (0.71–1.39)	0.84 (0.61–1.13) TFF	UVA	6/moderate
**Johnson/2015 [28]**	90	Retrospective/≥24	Melanoma	III-IV	IPI	NR/12	>1 month in 10 pts/high dose in 7 pts, NR in 5	IrAEs (100%)	1.06 (0.39–2.83)	-	UVA	8/low
**Ricciuti/2019 [22]**	650	Retrospective/NR	NSCLC	IV (100)	anti-PD-1/PD-L1 ± anti-CTLA-4	PDN/93	within 24 h of immunotherapy initiation/>10 mg vs. <10 mg	BMs (57%) BSC (43%) other (29%)	1.60 (1.07–2.39) * 0.91 (0.47–1.79)	1.40 (0.98–2) * 0.62 (0.33–1.17)	MVA	7/low
**Scott/2018 [20]**	210	Retrospective/NR	NSCLC	IV (100)	NIVO	PDN/66	concurrent/>10 mg	BMs (27%), BSC (39%) IrAEs (17%) other (17%)	2.3 (1.27–4.16) *	-	MVA	6/moderate
**Shafqat/2018 [36]**	157	Retrospective/6.7	Various	IV (100)	PEMBRO, NIVO or ATEZO	PDN/ 21	8.5 weeks (median)/NR	IrAEs (100%)	-	0.383 (0.16–0.918) *	MVA	6/moderate
**Sukari/2019 [31]**	168	Retrospective/26	Various	IV (100)	PEMBRO, NIVO	NR/77	NR/NR	IrAEs (100%)	0.81 (0.51–1.30)	-	MVA	8/low
**Taniguchi/2017 [27]**	201	Retrospective/NR	NSCLC	IV (100)	NIVO	NR/23	NR/(1.56-12-5)	Not specified (100%)	-	2.37 (1.44–3.74) *	MVA	6/moderate
**Weber/2009 [30]**	115	Randomized phase 2/14	Melanoma	III–IV	IPI	BUD/58	Baseline/NR	IrAEs (100%)	1.06 (0.66–1.7)	-	UVA	4 (Jadad)/low
**Zaragoza/2016 [29]**	58	Retrospective/33	Melanoma	IV (98.3)	IPI	NR/15	before week 1/NR	NR	1.28 (0.54–3.06)	-	MVA	8/low

*, statistically significant; ICIs, immune checkpoint inhibitors; PDN, prednisone; DEX, dexamethasone; methyl-P, methylprednisolone; BUD, budesonide; HR, hazard ratio; OS, overall survival; PFS, progression-free survival; NSCLC, non-small cell lung cancer; PEMBRO, pembrolizumab; NIVO, nivolumab; ATEZO, atezolizumab; DURVA, durvalumab; IPI, ipilimumab; TTF, time to treatment failure; TTNTD, time to next treatment or death; °, both for cancer-related and unrelated reasons; NOS, Nottingham–Ottawa Scale; NR, not reported; IPI, ipilimumab; ^, comparison of high vs low dose steroids; BM, brain metastases; BSC, best supportive care.

**Table 2:** Subgroup analysis for overall survival.

Subgroup Analysis	N of Studies/pts	HR (95% CI)	*p*	I^2^	Type of Analysis
Multi- vs. mono-centric studies • Multi-center • Single institution					
	7/2866	1.47 (1.25–1.72)	<0.01	0%	Random effect model
	9/1179	1.71 (1.18–2.46)	<0.01	75%	Random effect model
Type of agent • Anti-PD-L1 • Anti-CTLA-4					
	8/2540	1.50 (1.15–1.95)	<0.01	54%	Random effect model
	6/704	1.68 (0.97–2.92)	0.06	76%	Random effect model
Reason for steroid use • BSC • BMs • AEs					
	3/836	2.5 (1.41–4.43)	<0.01	76%	Random effect model
	3/1164	1.51 (1.22–1.87)	<0.01	49%	Random effect model
	9/926	1.08 (0.79–1.49)	0.62	48%	Random effect model
Number of patients • >100 • <100					
	10/3615	1.31 (1.05–1.64)	0.02	64%	Random effect model
	6/430	2.21 (1.44–3.41)	<0.01	47%	Random effect model
Type of analysis • UVA • MVA					
	4/548	1.49 (0.78–2.84)	0.23	79%	Random effect model
	12/3497	1.59 (1.28–1.97)	<0.01	52%	Random effect model
Quality of study • NOS score ≥7 • NOS score <7					
	8/2827	1.52 (1.16–1.99)	<0.01	66%	Random effect model
	8/1218	1.84 (1.07–3.17)	0.03	71%	Random effect model
Type of study • Retrospective • Prospective (1 study)					
	15/3930	1.59 (1.26–2)	<0.01	65%	Random effect model
	1/115	1.06 (0.66–1.7)	0.81	NA	Random effect model
Type of disease • NSCLC • melanoma					
	7/2944	1.62 (1.36–1.93)	<0.01	23%	Random effect model
	7/776	1.75 (1.07–2.88)	0.03	74%	Random effect model

BM, brain metastases; BSC, best supportive care; NSCL, non-small cell lung cancer; UVA, univariate analysis; MVA, multivariate analysis; NOS, Nottingham–Ottawa Scale; AEs, adverse events; PD-L1, programmed death ligand 1.

## References

[1] Signorelli D., Giannatempo P., Grazia G., Aiello M.M., Bertolini F., Mirabile A., Buti S., Vasile E., Scotti V., Pisapia P. (2019). Patients Selection for Immunotherapy in Solid Tumors: Overcome the Naïve Vision of a Single Biomarker. Biomed. Res. Int..

[2] Gandhi L., Rodríguez-Abreu D., Gadgeel S., Esteban E., Felip E., De Angelis F., Domine M., Clingan P., Hochmair M.J., Powell S.F. (2018). Pembrolizumab plus chemotherapy in metastatic non-small-cell lung cancer. N. Engl. J. Med..

[3] Paz-Ares L., Luft A., Vicente D., Tafreshi A., Gümüş M., Mazières J., Hermes B., Çay Şenler F., Csőszi T., Fülöp A. (2018). Pembrolizumab plus chemotherapy for squamous non-small-cell lung cancer. N. Engl. J. Med..

[4] Socinski M.A., Jotte R.M., Cappuzzo F., Orlandi F., Stroyakovskiy D., Nogami N., Rodríguez-Abreu D., Moro-Sibilot D., Thomas C.A., Barlesi F. (2018). Atezolizumab for first-line treatment of metastatic nonsquamous NSCLC. N. Engl. J. Med..

[5] Libert C., Dejager L. (2014). How Steroids Steer T Cells. Cell Rep..

[6] Bianchi M., Meng C., Ivashkiv L.B. (2000). Inhibition of IL-2-induced Jak-STAT signaling by glucocorticoids. Proc. Natl. Acad. Sci. USA.

[7] Chen X., Oppenheim J.J., Winkler-Pickett R.T., Ortaldo J.R., Howard O.M.Z. (2006). Glucocorticoid amplifies IL-2-dependent expansion of functional FoxP3 + CD4 + CD25 + T regulatory cells in vivo and enhances their capacity to suppress EAE. Eur. J. Immunol..

[8] Tetel M.J., de Vries G.J., Melcangi R.C., Panzica G., O’Mahony S.M. (2018). Steroids, stress and the gut microbiome-brain axis. J. Neuroendocr..

[9] Sica A., Mantovani A. (2012). Macrophage plasticity and polarization: In vivo veritas. J. Clin. Investig..

[10] Haanen J.B.A.G., Carbonnel F., Robert C., Kerr K.M., Peters S., Larkin J., Jordan K., ESMO Guidelines Committee (2017). Management of toxicities from immunotherapy: ESMO Clinical Practice Guidelines for diagnosis, treatment and follow-up. Ann. Oncol..

[11] Carbone D.P., Reck M., Paz-Ares L., Creelan B., Horn L., Steins M., Felip E., van den Heuvel M.M., Ciuleanu T.E., Badin F. (2017). First-line nivolumab in stage IV or recurrent non-small-cell lung cancer. N. Engl. J. Med..

[12] Reck M., Rodríguez-Abreu D., Robinson A.G., Hui R., Csőszi T., Fülöp A., Gottfried M., Peled N., Tafreshi A., Cuffe S. (2016). Pembrolizumab versus Chemotherapy for PD-L1-Positive Non-Small-Cell Lung Cancer. N. Engl. J. Med..

[13] Wolfe F., Caplan L., Michaud K. (2006). Treatment for rheumatoid arthritis and the risk of hospitalization for pneumonia: Associations with prednisone, disease-modifying antirheumatic drugs, and anti-tumor necrosis factor therapy. Arthritis Rheum..

[14] Lin R.J., Adelman R.D., Mehta S.S. (2012). Dyspnea in palliative care: Expanding the role of corticosteroids. J. Palliat. Med..

[15] Paulsen O., Klepstad P., Rosland J.H., Aass N., Albert E., Fayers P., Kaasa S. (2014). Efficacy of methylprednisolone on pain, fatigue, and appetite loss in patients with advanced cancer using opioids: A randomized, placebo-controlled, double-blind trial. J. Clin. Oncol..

[16] Ryken T.C., McDermott M., Robinson P.D., Ammirati M., Andrews D.W., Asher A.L., Burri S.H., Cobbs C.S., Gaspar L.E., Kondziolka D. (2010). The role of steroids in the management of brain metastases: A systematic review and evidence-based clinical practice guideline. J. Neurooncol..

[17] Horvat T.Z., Adel N.G., Dang T.O., Momtaz P., Postow M.A., Callahan M.K., Carvajal R.D., Dickson M.A., D’Angelo S.P., Woo K.M. (2015). Immune-related adverse events, need for systemic immunosuppression, and effects on survival and time to treatment failure in patients with melanoma treated with ipilimumab at memorial sloan kettering cancer center. J. Clin. Oncol..

[18] Leighl N., Gandhi L., Hellmann M.D., Horn L., Ahn M.-J., Garon E.B., Hui R., Ramalingam S.S., Zhang J., Lubiniecki G. (2015). Pembrolizumab for NSCLC: Immune-mediated adverse events and corticosteroid use. J. Thorac. Oncol..

[19] Arbour K.C., Mezquita L., Long N., Rizvi H., Auclin E., Ni A., Martínez-Bernal G., Ferrara R., Lai W.V., Hendriks L.E.L. (2018). Impact of baseline steroids on efficacy of programmed cell death-1 and programmed death-ligand 1 blockade in patients with non–small-cell lung cancer. J. Clin. Oncol..

[20] Scott S.C., Pennell N.A. (2018). Early Use of Systemic Corticosteroids in Patients with Advanced NSCLC Treated with Nivolumab. J. Thorac. Oncol..

[21] Fucà G., Galli G., Poggi M., Lo Russo G., Proto C., Imbimbo M., Ferrara R., Zilembo N., Ganzinelli M., Sica A. (2019). Modulation of peripheral blood immune cells by early use of steroids and its association with clinical outcomes in patients with metastatic non-small cell lung cancer treated with immune checkpoint inhibitors. ESMO Open.

[22] Ricciuti B., Dahlberg S.E., Adeni A., Sholl L.M., Nishino M., Awad M.M. (2019). Immune Checkpoint Inhibitor Outcomes for Patients with Non–Small-Cell Lung Cancer Receiving Baseline Corticosteroids for Palliative Versus Nonpalliative Indications. J. Clin. Oncol..

[23] Stroup D.F., Berlin J.A., Morton S.C., Olkin I., Williamson G.D., Rennie D., Moher D., Becker B.J., Sipe T.A.T.S. (2010). Meta-analysis of observational studies in epidemiology: A proposal for reporting. Meta-analysis Of Observational Studies in Epidemiology (MOOSE) group. JAMA.

[24] Sterne J.A., Hernán M.A., Reeves B.C., Savović J., Berkman N.D., Viswanathan M., Henry D., Altman D.G., Ansari M.T., Boutron I. (2016). ROBINS-I: A tool for assessing risk of bias in non-randomised studies of interventions. BMJ.

[25] Higgins J.P., Altman D.G., Gøtzsche P.C., Jüni P., Moher D., Oxman A.D., Savovic J., Schulz K.F., Weeks L., Sterne J.A. (2011). The Cochrane Collaboration’s tool for assessing risk of bias in randomised trials. BMJ.

[26] Hartling L., Milne A., Hamm M.P., Vandermeer B., Ansari M., Tsertsvadze A., Dryden D.M. (2013). Testing the Newcastle Ottawa Scale showed low reliability between individual reviewers. J. Clin. Epidemiol..

[27] Taniguchi Y., Tamiya A., Isa S.I., Nakahama K., Okishio K., Shiroyama T., Suzuki H., Inoue T., Tamiya M., Hirashima T. (2017). Predictive Factors for Poor Progression-free Survival in Patients with Non-small Cell Lung Cancer Treated with Nivolumab. Anticancer Res..

[28] Johnson D.B., Friedman D.L., Berry E., Decker I., Ye F., Zhao S., Morgans A.K., Puzanov I., Sosman J.A., Lovly C.M. (2015). Survivorship in Immune Therapy: Assessing Chronic Immune Toxicities, Health Outcomes, and Functional Status among Long-term Ipilimumab Survivors at a Single Referral Center. Cancer Immunol. Res..

[29] Zaragoza J., Caille A., Beneton N., Bens G., Christiann F., Maillard H., Machet L. (2016). High neutrophil to lymphocyte ratio measured before starting ipilimumab treatment is associated with reduced overall survival in patients with melanoma. Br. J. Dermatol..

[30] Weber J., Thompson J.A., Hamid O., Minor D., Amin A., Ron I., Ridolfi R., Assi H., Maraveyas A., Berman D. (2009). A randomized, double-blind, placebo-controlled, phase II study comparing the tolerability and efficacy of ipilimumab administered with or without prophylactic budesonide in patients with unresectable stage III or IV melanoma. Clin. Cancer Res..

[31] Sukari A., Nagasaka M., Alhasan R., Patel D., Wozniak A., Ramchandren R., Vaishampayan U., Weise A., Flaherty L., Jang H. (2019). Cancer Site and Adverse Events Induced by Immune Checkpoint Inhibitors: A Retrospective Analysis of Real-life Experience at a Single Institution. Anticancer Res..

[32] Chasset F., Pages C., Biard L., Roux J., Sidina I., Madelaine I., Basset-Seguin N., Viguier M., Madjlessi-EzrA N., Schneider P. (2015). Single-center study under a French Temporary Authorization for use (TAU)) protocol for ipilimumab in metastatic melanoma: Negative impact of baseline corticosteroids. Eur. J. Dermatol..

[33] Acharya S., Mahmood M., Mullen D., Yang D., Tsien C.I., Huang J., Perkins S.M., Rich K., Chicoine M., Leuthardt E. (2017). Distant intracranial failure in melanoma brain metastases treated with stereotactic radiosurgery in the era of immunotherapy and targeted agents. Adv. Radiat. Oncol..

[34] Dumenil C., Massiani M.A., Dumoulin J., Giraud V., Labrune S., Chinet T., Giroux Leprieur E. (2018). Clinical factors associated with early progression and grade 3–4 toxicity in patients with advanced non-small-cell lung cancers treated with nivolumab. PLoS ONE.

[35] Faje A.T., Lawrence D., Flaherty K., Freedman C., Fadden R., Rubin K., Cohen J., Sullivan R.J. (2018). High-dose glucocorticoids for the treatment of ipilimumab-induced hypophysitis is associated with reduced survival in patients with melanoma. Cancer.

[36] Shafqat H., Gourdin T., Sion A. (2018). Immune-related adverse events are linked with improved progression-free survival in patients receiving anti-PD-1/PD-L1 therapy. Semin. Oncol..

[37] Hendriks L.E.L., Henon C., Auclin E., Mezquita L., Ferrara R., Audigier-Valette C., Mazieres J., Lefebvre C., Rabeau A., Le Moulec S. (2019). Outcome of Patients with Non–Small Cell Lung Cancer and Brain Metastases Treated with Checkpoint Inhibitors. J. Thorac. Oncol..

[38] Bertrand A., Kostine M., Barnetche T., Truchetet M.E., Schaeverbeke T. (2015). Immune related adverse events associated with anti-CTLA-4 antibodies: Systematic review and meta-analysis. BMC Med..

[39] Gandhi S., Pandey M., Ammannagari N., Wang K., Vona K.L., Nestico J., Hamad L., Dy G.K., Ernstoff M.S. (2017). Clinical and biochemical parameters as predictors of response to checkpoint inhibitors (CPI): A single institution experience. J. Clin. Oncol..

[40] Maxwell R., Luksik A.S., Garzon-Muvdi T., Hung A.L., Kim E.S., Wu A., Xia Y., Belcaid Z., Gorelick N., Choi J. (2018). Contrasting impact of corticosteroids on anti-PD-1 immunotherapy efficacy for tumor histologies located within or outside the central nervous system. Oncoimmunology.

[41] Xing K., Gu B., Zhang P., Wu X. (2015). Dexamethasone enhances programmed cell death 1 (PD-1) expression during T cell activation: An insight into the optimum application of glucocorticoids in anti-cancer therapy. BMC Immunol..

[42] Greenstein S., Ghias K., Krett N.L., Rosen S.T. (2002). Mechanisms of glucocorticoid-mediated apoptosis in hematological malignancies. Clin. Cancer Res..

[43] Brahmer J.R., Lacchetti C., Schneider B.J., Atkins M.B., Brassil K.J., Caterino J.M., Chau I., Ernstoff M.S., Gardner J.M., Ginex P. (2018). Management of immune-related adverse events in patients treated with immune checkpoint inhibitor therapy: American society of clinical oncology clinical practice guideline. J. Clin. Oncol..

